# Induction of the Antiviral Immune Response and Its Circumvention by Coronaviruses

**DOI:** 10.3390/v12091039

**Published:** 2020-09-18

**Authors:** Ping Liu, Yan Hong, Bincai Yang, Prasha Shrestha, Nelam Sajjad, Ji-Long Chen

**Affiliations:** 1 Key Laboratory of Fujian-Taiwan Animal Pathogen Biology, College of Animal Sciences (College of Bee Science), Fujian Agriculture and Forestry University, Fuzhou 350002, China; pingliu2010@163.com (P.L.); hongyan9853@163.com (Y.H.); Yangbincai1018yy@163.com (B.Y.); prasha.shrestha@yahoo.com (P.S.); neelamsajjad2@gmail.com (N.S.); 2 CAS Key Laboratory of Pathogenic Microbiology and Immunology, Institute of Microbiology, Chinese Academy of Sciences (CAS), Beijing 100101, China

**Keywords:** coronaviruses, viral tissue tropism, antiviral immune response, interferon signaling, immune evasion

## Abstract

Some coronaviruses are zoonotic viruses of human and veterinary medical importance. The novel coronavirus, severe acute respiratory symptoms coronavirus 2 (SARS-CoV-2), associated with the current global pandemic, is characterized by pneumonia, lymphopenia, and a cytokine storm in humans that has caused catastrophic impacts on public health worldwide. Coronaviruses are known for their ability to evade innate immune surveillance exerted by the host during the early phase of infection. It is important to comprehensively investigate the interaction between highly pathogenic coronaviruses and their hosts. In this review, we summarize the existing knowledge about coronaviruses with a focus on antiviral immune responses in the respiratory and intestinal tracts to infection with severe coronaviruses that have caused epidemic diseases in humans and domestic animals. We emphasize, in particular, the strategies used by these coronaviruses to circumvent host immune surveillance, mainly including the hijack of antigen-presenting cells, shielding RNA intermediates in replication organelles, 2′-O-methylation modification for the evasion of RNA sensors, and blocking of interferon signaling cascades. We also provide information about the potential development of coronavirus vaccines and antiviral drugs.

## 1. Introduction

Coronaviruses cause highly contagious diseases in both humans and animals and have led to severe epidemics that have caused major public health threats, such as the severe acute respiratory symptom (SARS) outbreak in 2002−2003 [1], and Middle East respiratory syndrome coronavirus (MERS), which emerged in 2012 [2]. At the very beginning of this new decade, a novel coronavirus, severe acute respiratory symptoms coronavirus 2 (SARS-CoV-2), emerged and caused a catastrophic pandemic of respiratory illness worldwide. During the period from December 2019 to 30 August 2020, the pandemic caused by the new coronavirus SARS-CoV-2 spread to 216 countries worldwide, infecting more than 24 million individuals, leading to 838,360 deaths, and severely crippling the worldwide economy [3]. Currently, COVID-19 is still spreading worldwide at unprecedented speed.

Interferon (IFN) production is a fundamental process involved in the innate immune response to viral infection. These soluble antiviral cytokines can induce upregulation of an array of intracellular effectors of interferon-stimulated genes (ISGs) through the Janus kinase/signal transducer and activator of transcription (JAK/STAT) signal pathways, such as Mx proteins, protein kinase PKR, and ISG15, which have antiviral activity as they halt viral replication and dissemination of infected cells [4]. Thus, before effective adaptive immune responses are initiated, the IFN-mediated innate immune response plays a critically important role in eliminating virus invasion and protecting tissue damage and inflammation at the early phase of infection [5]. However, recent studies revealed that excessive expression of type I and III IFNs might disrupt the repair of lung epithelial tissue during respiratory viral infection [6,7,8]. Indeed, coronaviruses are known to efficiently circumvent the host immune systems, and IFN production can barely be detected during the early phase of coronavirus infection [9,10]. Compared with SARS and MERS, COVID-19 patients have extended incubation periods without apparent symptoms, which subsequently contribute to the cytokine storm, damaging inflammation, and other severe complications [11,12]. On the other hand, coronaviruses might have evolved to escape innate immune responses by their infected hosts [13]. It has to be mentioned that IFN-antagonistic functions affect viral virulence to a considerable extent and might be associated with interspecies transmission of emerging and reemerging viruses [14]. Therefore, in this review, we summarize antiviral immunity in the respiratory and intestinal tracts against coronaviruses that have been reported to cause epidemics in both humans and domestic animals. In particular, we highlight how these different coronaviruses exploit multiple strategies to evade immune surveillance exerted by the host.

## 2. Coronavirus Taxonomy and Viral Tissue Tropism

Coronaviruses, members of the family Coronaviridae, have positive-stranded RNA genomes, and their virions are roughly spherical and moderately pleomorphic with an average diameter of 60−220 nm. Their genome range is approximately 26–33.5 Kb in length with a 5′-cap and 3′-polyadenylated tail displayed as ORF1a-ORF1b-S-ORF3-E-M-N in order, with both termini flanked and untranslated regions [15]. The 5′-proximal two-thirds of the genome contain a large replicase polyprotein, of which ORF1a encodes a large polyprotein, pp1a, and ORF1b expresses the pp1ab fusion protein using ribosomal frameshifting. These polyproteins can be proteolytically cleaved to 16 nonstructural proteins (nsp) from nsp1 to nsp16 [16].

To date, more than 30 coronaviruses have been identified to infect various species including humans, pigs, casts, dogs, horses, poultry, camels, and other wild animals [17,18]. Wild animals, such as bats and rodents, are the natural reservoirs of coronaviruses, and they transmit them to the intermediate hosts of camelids, civets, dromedary camels, pangolins, or domestic animals. They sometimes are eventually transmitted to the human population, resulting in severe epidemics in humans, such as SARS and MERS [19]. Recently, a pneumonia outbreak by SARS-CoV-2 was thought to have originated in bats [20]. This novel emerging coronavirus can efficiently replicate in ferrets and cats, but it replicates poorly in dogs, pigs, chickens, and ducks [18]. Together, these data show that some coronaviruses are critical zoonotic viruses that can cause public health problems for humans, and they also notoriously affect the agricultural industry. Based on the genotypic and serological characterization of coronaviruses, they are divided into four main genera: (a) Alphacoronaviruses, which mainly include human CoV-229E, human CoV-NL63, porcine epidemic diarrhea virus (PEDV), and transmissible gastroenteritis virus (TGEV); (b) Betacoronaviruses, which mainly include severe acute respiratory symptoms coronavirus (SARS-CoV), Middle East respiratory syndrome coronavirus (MERS-CoV), severe acute respiratory symptoms coronavirus 2 (SARS-CoV-2), mouse hepatitis virus (MHV), and bovine CoV (BCoV); (c) Gammacoronaviruses are related to avian diseases, including infectious bronchitis virus (IBV) and turkey blue comb virus (TBV); (d) Deltacoronaviruses. Generally, Alpha and Betacoronaviruses invade mammalian hosts, whereas Gammacoronaviruses infect avian hosts. The Deltacoronavirus genus is a novel group of coronaviruses identified from pigs, one of which, porcine Deltacoronavirus (PDCoV), emerged in the USA, Korea, and China, resulting in huge economic losses in the pig industry [21,22,23].

The envelope spike (S) protein of coronavirus is a large transmembrane glycoprotein that is involved in receptor binding, membrane fusion, and entry into the host cells. The S protein interacts with unique receptors in susceptible target cells of the host species and presents distinct viral tissue tropism [24]. Coronaviruses infecting mammalian animals principally cause histopathological lesions in the intestinal and respiratory tracts, but in some cases, they cause central neurologic illness and hepatitis. In humans, coronaviruses have been proven to be mainly associated with respiratory illnesses [17]. Specifically, the mouse hepatitis coronavirus and feline coronavirus can cause pneumonia and gastrointestinal infections as well as hepatitis and neurologic disorders. It has been indicated that the surface angiotensin-converting enzyme 2 (ACE2) receptor is required for host cell entry of both SARS-CoV and SARS-CoV-2 viruses [25]. The dipeptidyl peptidase-4 (DPP4) receptor is associated with MERS-CoV in respiratory organs [26], whereas aminopeptidase N (APN) is the functional receptor for the entry of enteric coronaviruses, such as PEDV and PDCoV [27,28,29]. Besides the ACE2 receptor, the transmembrane serine protease 2 (TMPRSS2) has been shown to activate the S protein for membrane fusion in SARS-CoV and SARS-CoV-2 infection [30]. Notably, ACE2 and TMPRSS2 are also highly expressed in the small intestine of the gut [31,32]. More than 60% of patients infected with SARS-CoV-2 present with intestinal disorders such as diarrhea, nausea, and vomiting, particularly those present with severe clinical disease. Thus, it has been suggested that the digestive system is a potential route for SARS-CoV-2 infection [33,34].

## 3. Antiviral Immune Responses to Coronavirus Infection

**Figure 1:**
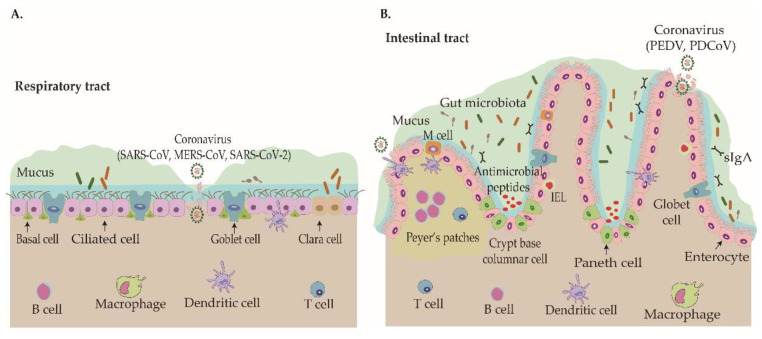
Architecture of the mucosal epithelial barrier in the respiratory and intestinal tracts that guards against viral invasion. (**A**). The airway epithelium is composed of ciliated cells, goblet cells, basal cells, and Clara cells. The mucus on the epithelial surface is the first barrier against human infection by coronaviruses, such as SARS-CoV, MERS-CoV, and the novel emerging coronavirus, SARS-CoV-2. The mucins secreted by goblet cells on the epithelial surface include two layers, a viscous layer on top and a periciliary layer below. The innate immune cells in the submucosal layer such as dendritic cells and macrophages are involved in controlling viral infection. (**B**) Enteric coronaviruses, such as PEDV and PDCoV, principally infect swine by causing histopathological lesions in the intestinal tract. In spite of their similar histological structures, there are substantial differences in the functional purposes and internal environments of the gut and respiratory tract. The mucus of the intestinal tract mainly consists of MUC2 mucin, antimicrobial peptides, and secreted IgA (sIgA) produced by goblet cells, Paneth cells, and plasma cells, respectively. In particular, the commensal bacterial communities resident in the mucus of the gut are involved in various physiological processes that modulate the homeostasis of mucosal immunity. In addition, the intraepithelial lymphocytes (IELs) are located between intestinal epithelial cells, and these cells constitute a large and highly conserved T cell compartment. Intestinal microfold cells (M cells) are only found in the gut-associated lymphoid tissues (GALT) of Peyer’s patches in the intestinal tract, and they are unique antigen-presenting cells that are important for the initiation of mucosal immune responses. The diverse immune cells reside in the *lamina propria* and mainly include B cells, T cells, dendritic cells, and macrophages. These immune cells interact with the epithelium to detect invading pathogens.

The respiratory and intestinal tracts are crucial opening windows of the host that are exposed to various toxic antigens and pathogenic agents. A set of elaborate immune mediators guard the tissues against invading pathogens at these luminal surfaces. Both tracts are essential constituents of the mucosal immune system, and the mucosal barrier structure and defense mechanisms against pathogens are quite similar (Figure 1).

Interestingly, the homeostasis of the complex mucosal system is modulated by gut microbiota and immune cells, which form an interactive network via the “gut−lung” axis [35]. Most recently, studies have suggested that the gut microbiota composition might underlie the predisposition of normal individuals to severe COVID-19 [36].

### 3.1. The Protective Roles of the Mucus Barrier

The mucus gel forms a continuous layer on the mucosal epithelial surface of the respiratory and intestinal tracts (Figure 1) and provides an essential first defense barrier against aggressive luminal factors and pathogens [37]. Mucins secreted by goblet cells are the main component of airway/intestinal mucus. Respiratory mucus can be divided into two discreet layers, a viscous layer on top and a periciliary layer (PCL) below. The viscous layer comprises the secreted mucins *MUC5AC* and *MUC5B*, whereas the PCL contains membrane-tethered mucins such as *MUC1*, *MUC4*, and *MUC16* [38]. Mucins are mainly glycoproteins. Specifically, the membrane-tethered mucins are characterized by a large glycosylated extracellular domain that possibly provides binding sites for potential pathogens and a cytoplasmic tail that may induce signaling cascades [39]. Previous studies have shown that *MUC5AC* and *MUC1* are key, dynamic components of innate immunity that limit the severity of influenza virus infection, suggesting that mucins are an essential defense barrier against virus invasion [38,40]. Interestingly, sialic acid is a key oligosaccharide component of mucin, and previous studies have indicated that influenza virus, as well as some coronaviruses such as BCoV and MERS-CoV, could interact with different sialic acid residues to modulate the infection efficiency [40,41]. Moreover, the number of goblet cells was shown to be depleted or significantly decreased in the small intestine during the early phase of PEDV infection [42], but studies focused on the interplay between different phenotypes of mucins and animal coronaviruses are still lacking. Whether the glycoprotein of SARS-CoV-2 can interact with the extracellular domain of cell-surface mucins also needs to be experimentally elucidated.

### 3.2. Innate Antiviral Signaling

Innate immunity signaling cascades are activated by recognition of the pathogen-associated molecular patterns (PAMPs) of the invading virus by pattern-recognition receptors (PRRs). The PRRs involved in the detection of viral pathogens mainly include toll-like receptors (TLRs), retinoic acid-inducible gene (RIG) type I like receptors (RLRs), C-type lectin-like receptors (CLRs), and nucleotide-binding oligomerization domain-like receptors (NLRs) [43]. It is known that PRRs are widely distributed on the plasma membrane, endosomal membranes, and in the cytoplasm of different host cells to detect foreign substances [44]. PAMPs of coronaviruses, such as amino acids, double-stranded RNA (dsRNA), single-stranded RNA (ssRNA), and glycoproteins, can be recognized during viral infection and replication via TLRs and RLRs, resulting in the activation of innate immune signaling cascades [13,45].

**Figure 2:**
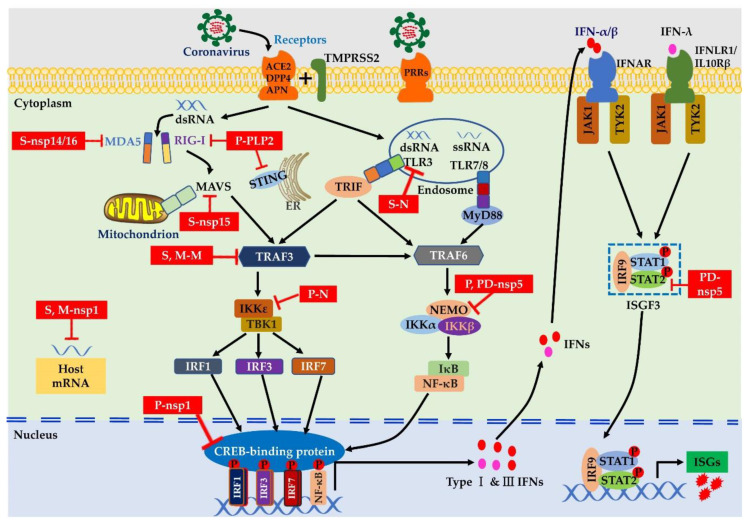
Schematic diagram of the antiviral immune response and evasion mechanism of coronaviruses. Coronaviruses are internalized into susceptible target cells by the fusion of viral and cellular membranes with unique receptors, such as ACE2, DPP4, and APN, and the RNA of the viral genome is released into the cytosol. SARS-CoV and SARS-CoV-2 exploit the serine protease TMPRSS2 for spike protein priming. The virion and the pathogen-associated molecular patterns (PAMPs) of coronavirus can be recognized by immune sensors called pattern-recognition receptors (PRRs), such as toll-like receptors (TLRs) and cytoplasm retinoic acid-inducible gene (RIG) type I like receptors (RLRs) (RIG-I/MDA5). The extracellular membrane of TLRs (TLR2/4) and endosome TLRs (TLR3/7/8) are widely expressed in epithelial cells and dendritic cells. The PAMPs of coronaviruses induce the interferon (IFN) signaling pathway for antiviral innate immune responses. RIG-I/MDA5 conveys signals through mitochondrial antiviral-signaling protein (MAVS), while TLRs signal through TIR-containing adapter protein inducing IFN-β/myeloid differentiation factor 88 (TRIF/MyD88). TNF receptor-associated factor 3 (TRAF3) activates tank-binding kinase 1/IκB kinase epsilon (TBK1/IKKε), while TRAF6 signal transduction requires activation of the IKK complex. Activated transcription factors are translocated into the nucleus to promote Type I and III IFN expression. IFNs are secreted into the extracellular space and bound to their cognate receptors IFNAR and IFNLR (IFNLR1 and IL10Rβ) to activate downstream the Janus kinase/signal transducer and activator of transcription (JAK/STAT) signaling, followed by nuclear localization of the interferon-stimulated gene factor 3 (ISGF3) complex and expression of numerous interferon stimulating genes (ISGs), leading to the establishment of an antiviral state. Suppression of IFN signal pathways by coronaviruses and their antagonists is shown in red boxes. S, severe acute respiratory symptom coronavirus (SARS-CoV); M, Middle East respiratory syndrome coronavirus (MERS-CoV); P, porcine epidemic diarrhea virus (PEDV); PD, porcine Deltacoronavirus (PDCoV); ACE2, angiotensin-converting enzyme 2; DPP4, dipeptidyl peptidase-4; APN, aminopeptidase N; TMPRSS2, transmembrane serine protease 2.

TLRs are the best-characterized membrane-bound PRRs, and they are expressed on the cellular membrane and endomembrane of various cells, including epithelial cells, fibroblasts, macrophages, dendritic cells, B cells, and some T cells [46]. TLRs 2 and 4 are expressed on the surface of cells and are capable of detecting glycoproteins of viral PAMPs (Figure 2). They have been identified as potential receptors for S protein that induce the host’s innate immune response [47].

In addition, TLR4 expressed on the lung epithelium may act as a cofactor, affecting viral entry [48], and it may play a protective role in pathogenesis after MHV-infection in a mouse model [49]. TLRs 3, 7, 8, and 9 are distributed in the cytosol and involved in sensing endosomal nucleic acid of RNA intermediates. It has been shown that TLR3 signaling via TIR-containing adapter protein inducing IFN-β (TRIF) adaptor molecules induces an effective host cell-intrinsic antiviral defense against lethal SARS-CoV infection [50]. In contrast, TLRs 7 and 8 of plasmacytoid dendritic cells (pDCs) can recognize the ssRNA of viruses in the lysosome and activate IFN signaling via the adaptor protein myeloid differentiation factor 88 (MyD88) during the viral infection [51]. A previous study indicated that pDCs control the fast replication of MHV through TLR7-mediated type I IFN production [52]. Interestingly, a recent study using a bioinformatics approach demonstrated that the SARS-CoV-2 genome is characterized by more ssRNA, which might be detected by TLR7/8 and could possibly trigger a robust proinflammatory response [53]. Nevertheless, whether pDCs are essential for controlling viral infection still needs to be tested in SARS-CoV-2 patients. Moreover, the enteric coronavirus PEDV induces nuclear factor-kappa B (NF-κB) activation by TLR 2, 3, and 9 pathways, indicating that the innate immune sensors can recognize the PEDV surface glycoproteins as well as RNA intermediates in the cytoplasm [54].

In contrast, RLRs, mainly including MDA5 and RIG-I, are cytoplasmic recognition molecules that sense RNA viruses in many cell types [55]. Recognition of RNA viruses by RLRs induces the activation of several transcriptional factors, mainly including interferon regulatory factor (IRF) 1, IRF3, IRF7, and NF-κB. The transcriptional factors of IRFs 1/3/7 could trigger the transcription of Type I IFNs (IFN-α/β) and Type III IFNs (IFN-λ) [45], while NF-κB mediates the transcription of proinflammatory cytokines [56]. For instance, MHV can be detected by both MDA5 and RIG-I, which induces Type I IFN production in oligodendrocytes via the activation of the transcriptional factor IRF3 [57]. Meanwhile, infection with MHV activates MDA5 signaling, resulting in decreased levels of proinflammatory cytokines, which effectively reduces the severity of MHV-induced pathogenesis [58]. Nevertheless, SARS-CoV infection causes temporal and spatial activation of the transcriptional factor NF-κB, which leads to transcription of proinflammatory cytokines at 12 h postinfection, whereas transcription of Type I IFNs by IRF3/IRF7 is delayed until 48 h postinfection [59]. SARS-CoV-2 has been proven to share approximately 80% of the genome with SARS-CoV, and almost all of the encoded proteins of SARS-CoV-2 are highly homologous to SARS-CoV proteins [60]. Thus, SARS-CoV-2 may produce similar RNA intermediates that trigger TLR and RIG-I signaling during the viral life cycle.

### 3.3. Cytokines Involved in Antiviral Innate Responses

The epithelium and immune cells in the respiratory and intestinal tracts detect the invading virus via innate immune sensors and trigger a series of well-coordinated immune responses, leading to the production of proinflammatory cytokines, chemokines, and ISGs, which protect the host cells from viral infection during the early phase. However, patients with severe disease who are infected with highly pathogenic viruses, such as influenza virus and SARS-CoV-2, normally have a dysfunctional proinflammatory response, leading to a cytokine storm and immunopathological changes [36,61].

#### 3.3.1. Interferons

IFNs are crucial cytokines in antiviral host defenses [62]. Type Ι IFNs can be expressed in many diverse cell types, whereas type III IFNs are mainly produced in epithelial cells and dendritic cells [63]. Recently, it has become clear that despite the similarities between type Ι IFN and type III IFN signaling cascades, type III IFNs seem to specifically protect the epithelial cells from pathogen attacks and form a local special defense in the early stage of attack [6,64]. When the first activation of the IFN-λ machinery only weakly disseminates the locally invading virus, the type Ι IFN apparatus is mobilized to mediate a systemic antiviral immune response [65]. However, highly contagious coronaviruses have developed strategies to block IFN signaling. For instance, SARS-CoV can efficiently suppress IFN expression by its structural and nonstructural proteins, which has been shown to prevent the innate response of IFNs in cultured cells or SARS patients [66,67]. Inadequate or delayed IFN responses may explain the progressive increase in viral replication and immunopathology in SARS patients [68]. However, some studies have indicated that SARS-CoV-2 initiates a robust IFN response in patients compared with SARS-CoV cases [12,69]. Indeed, IFN-α/β and IFN-λ have been recommended as potential therapeutic drugs to prevent and treat SARS-CoV-2 infection [70,71,72]. Notably, excessive and prolonged IFN expression might lead to deleterious proinflammatory responses and may aggravate viral infection by disrupting the lung epithelial barrier [7,8]. Thus, the dose, frequency, and duration of IFN therapy should be considered carefully before use in clinical practice against viral infection.

#### 3.3.2. Proinflammatory Cytokines

SARS-CoV infection induces upregulation of proinflammatory cytokines/chemokines, such as interleukin (IL)-1β, IL-6, IL-8, IL-12, monocyte chemotactic protein 1 (MCP-1), and IFN-γ inducible protein (IP-10), in the tissues and serum during the first two weeks of illness onset [73]. Similarly, most of the patients infected with severe SARS-CoV-2 exhibit elevated levels of numerous proinflammatory cytokines in serum, including IL-1β, IL-1Ra, IL-2, IL-6, IL-8, IL-10, granulocyte colony-stimulating factor (G-CSF), granulocyte-macrophage colony-stimulating factor (GM-CSF), IP-10, macrophage inflammatory protein-1 alpha (MIP-1α), and TNF-α [12,74]. However, these proinflammatory cytokines were not significantly increased in patients with mild COVID-19 [75]. Thus, it is important to understand the dynamic changes in the proinflammatory response at the early stage of SARS-CoV-2 infection. In this context, Ong et al. conducted daily transcriptional profiling of whole blood from COVID-19 patients, and the data revealed that the early immune response was highly dynamic during COVID-19 progression. Meanwhile, the increased expression of IL-1 and its associated proinflammatory pathway might be correlated with severe respiratory disease [69]. To better understand the modulation of the immune response in target tissues of COVID-19 patients, a previous study analyzed immune signatures using bronchoalveolar lavage collected from COVID-19 patients. The results indicated that COVID-19 patients display robust innate immune responses with chemokine-dominated hypercytokinemia, such as elevated expression of chemokine (CXC motif) ligand 17 (CXCL17), CXCL8, CXCL1, CXCL2, and chemokine (CXC motif) receptor 2 (CXCR2), suggesting the importance of the recruitment of neutrophils and monocytes in controlling viral infection [68].

#### 3.3.3. Interferon-Stimulated Genes (ISGs)

IFNs activate the kinases of the JAK family and phosphorylate several members of the STAT family of transcription factors in an autocrine and paracrine manner, resulting in the expression of numerous ISGs that establish an “antiviral state” in both infected host cells and the surrounding uninfected cells [56,76]. This state can efficiently inhibit viral infection and replication, and it also simultaneously induces priming of the adaptive immune responses which, in most cases, eventually clears the virus from the infected host. ISGs known to exert direct antiviral activity are upregulated during viral infection, such as IFN-induced proteins with IFN-induced transmembrane proteins (IFITMs), tetratricopeptide repeats (IFITs), and ISG15. These ISGs have broad-spectrum antiviral functions [77,78]. It has been demonstrated that IFITMs can inhibit cellular entry of MERS-CoV [79] and SARS-CoV [80]. SARS-CoV-2 induces a robust IFN response and substantially increases the expression of IFITM2 and IFITM3 [68]. Despite the upregulation of ISGs in COVID-19 patients, there is no clear correlation between the observed ISG upregulation and disease severity [81]. The potential ISGs exert protective functions against SARS-CoV-2 infection, which may explain the lower proportion of severe cases and the lower case-fatality rate in COVID-19 patients compared with SARS cases [82]. However, a high viral load has been detected at the very early phase of SARS-CoV-2 infection after symptom onset [83], suggesting that the novel emerging coronavirus might exploit unique strategies to evade host innate immunity.

## 4. Strategies of Coronaviruses to Circumvent Host Immune Surveillance

Coronaviruses are well known to circumvent the innate immune response, particularly during the early phase of infection. It has been shown that IFN production can barely be detected during the first hours of infection, which greatly facilitates the establishment of productive viral replication [9,10]. Coronaviruses employ multiple strategies to evade the host’s innate immune response, for instance, hijack of the antigen-presenting cells, formation of a replication organelle for RNA synthesis, modification at the 5′end with 2′-O methylation of viral RNA, and inhibition of IFN signaling cascades.

### 4.1. Hijack of the Antigen-Presenting Cells by Coronavirus Spike Glycoprotein

Besides the receptors on the membranes of susceptible target cells, the spike glycoprotein can also bind to cellular factors on antigen-presenting cells such as dendritic cells (DCs) and microfold cells. The pathogens bind to factors on DCs, which can aid in their spread to infect other individuals [84]. DCs can capture antigens directly in the lumen by forming transepithelial dendrites. These antigen-bearing DCs can migrate to the adjacent lymph nodes and present the antigens to T cells or B cells for further activation of cellular and humoral immune responses [85]. DCs possess high levels of TLRs, and they also express CLRs, such as DC-specific intercellular adhesion molecule 3-grabbing nonintegrin (DC-SIGN). Viral glycoproteins interact with DC-SIGN, which helps viruses to break through the epithelial barrier. Thus, they act as a “Trojan Horse”, allowing the virus to evade antiviral immune responses, resulting in virus dissemination in the submucosal layer of mucosal tissues [86]. It has been reported that nasal dendritic cells are hijacked by PEDV, and then the virus is transferred to CD3^+^ T cells in the submucosa [87]. DCs are also crucial target cells of MERS-CoV that participate in viral replication and dissemination [88]. Additionally, the SARS-CoV spike glycoprotein interacts with DC-SIGN and contributes to the low production of antiviral cytokines such as IL-12 and IFNs, which might help the virus evade the innate immune system [84]. The mentioned coronaviruses bind to the antigen-presenting cells, such as DCs, using the unique glycoproteins on their envelope, which facilitate the virus in breaking through the mucosal barrier and avoiding detection by the PRRs on the cellular membrane of the epithelium. To date, little information is available about the roles of DCs and pDCs in SARS-CoV-2 infection. Thus, further investigation is required to elucidate whether the DCs could mediate SARS-CoV-2 transmission to target cells.

### 4.2. Shielding RNA Intermediates in Replication Organelles

Coronaviruses produce RNA intermediates such as dsRNA and ssRNA during replication in host cells. In contrast, mammalian host cells cannot produce such RNA species from RNA templates. These RNA intermediates can be detected through innate immune sensors, leading to antiviral effector activation. However, the RNA intermediates can shield their features in the replication organelles (ROs) from PRR detection, thereby suppressing the intracellular innate immune responses in the cytoplasm [89]. The intracellular membrane ROs are convoluted double-membrane vesicles formed by the host endoplasmic reticulum, which are thought to be associated with the viral synthesis of positive-stranded RNA [90]. Coronaviruses, like all positive-stranded RNA viruses, hijack endoplasmic reticulum membranes to form their headquarters for viral RNA synthesis exclusively in the cytosol. A previous study showed that nonstructural proteins (nsp) 3, 4, and 6 of SARS-CoV are specific hydrophobic viral proteins that contain transmembrane domains for the formation of double-membrane vesicles [91]. Similarly, MERS-CoV nsp3 and nsp4 can also mimic the formation of ROs, shielding their viral dsRNA from innate immune sensors in the cytosol, such as MDA5 and RIG-I [92]. Compared with the formation of convoluted membranes of Alpha and Betacoronaviruses, the specific formation of a zippered endoplasmic reticulum with tethered double-membrane spherules was observed in the Gammacoronavirus avian infectious bronchitis virus [93] and porcine Deltacoronavirus [94,95]. However, there is still little known about the detail of the interplay between ROs and the antiviral defense. Most of the studies on such organelles of RNA viruses have involved cells, and thus, it is important to study the presence of ROs during coronavirus infection in hosts. In addition, how the RNA intermediates are released from ROs, and whether the ROs can be dissolved or still exist in the cytosol after viral RNA synthesis, need to be further elucidated.

### 4.3. Coronaviruses Suppress Innate Immune Signaling

Coronaviruses encode multiple proteins as antagonists of IFN signaling that impede or delay the IFN signal cascades and expression of ISGs and, consequently, contribute to viral pathogenesis [65]. Some reports have shown that SARS-CoV encodes at least 11 viral proteins as IFN antagonists [96], and more than 10 viral proteins of PEDV have an inhibitory function that is involved in the regulation of IFN expression [97]. Indeed, various coronavirus proteins exploit diverse strategies to suppress the innate immune signaling pathways, of which the viral protein functions of nonstructural proteins such as nsp1, nsp3, nsp5, and nsp16 are emphasized in this review (Figure 2).

#### 4.3.1. Shut-Off of Host Protein Synthesis

Some viruses and their protein components can halt cellular protein expression in the host and simultaneously utilize the cellular translation apparatus for their use. Coronaviruses use a combined method to inhibit host protein synthesis at the levels of transcription and translation. Nsp1 is the 5′-terminal subunit for the replication of polyproteins in coronaviruses [98], and it is one of the most divergent genes among the four different coronavirus genera. Only the genomes of Alpha and Betacoronaviruses encode nsp1, and the genomes of Gammacoronaviruses and Deltacoronaviruses lack the nsp1 gene. Specifically, the nsp1 gene of Betacoronaviruses is much larger than that of Alphacoronaviruses [97,99]. Coronavirus nsp1 could bind to the cellular molecules of the translation apparatus and prevent the translation of host mRNAs. For example, SARS-CoV nsp1 is localized exclusively in the cytoplasm and directly binds to the 40S ribosomal subunit of ribosomes, resulting in inhibition of the protein and translation of host mRNA [100]. However, nsp1 of MERS-CoV is distributed in both the nucleus and the cytoplasm and targets the translational competence of host mRNAs selectively for inhibition and degradation [101]. Nsp1 of PEDV, the enteric coronavirus, can degrade the cAMP responsive element binding (CREB)-binding protein (CBP) by the proteasome, thus preventing the formation of IRF3 and the CBP enhancer complex and, consequently, resulting in inhibition of IFN-β production [45,97].

#### 4.3.2. Manipulation of the Ubiquitin Process

Ubiquitin is a key regulatory factor that corrects the function of cellular processes mediated by linkage of the chain, of which K48-linked ubiquitin chains contribute to degradation. It has been revealed that viruses exploit several strategies to interfere with the ubiquitin cellular system [102]. Nsp3 of coronaviruses expresses papain-like protease (PLpro), which is a kind of deubiquitinase (DUB) [103]. SARS-CoV PLpro has deubiquitinating activity and can potentially deconjugate cellular substrates to inhibit the ubiquitin-mediated signal pathway [103]. The DUB activity of SARS-CoV PLpro is manifested in the K48 lysine-linked polyubiquitin mediated degradation of the target protein through the ubiquitin−proteasome pathway [102]. In contrast, MERS-CoV PLpro shows broad linkage specificity for the cleavage of polyubiquitin chains [102], and PLpro can block host antiviral responses by inhibiting the expression of IFN-β [104]. Additionally, the enteric coronavirus PEDV PLP2 shows deubiquitinating activity on the RLRs of RIG-I and STING, leading to the evasion of innate immune signaling routes [105]. PLpros from other coronaviruses, such as HCoV-NL63 PLP2, TGEV PLP1, and MHV PLP2, exert similar inhibitory functions to that of IFN expression [106]. This indicates that coronavirus PLpro is highly conserved in viral evolutionary processes and is recognized as an attractive antiviral drug target.

#### 4.3.3. Cleavage of Innate Immune Factors

The 3-chymotrypsin-like cysteine protease (3CLpro, also called Mpro) of coronavirus is the main proteinase that controls the activities of the viral replication complex. This protease often exerts unique functions to support virus immune evasion [107]. For example, PEDV 3CLpro is used for cleaving cellular substrates to promote viral infection and inhibit the innate immune response through cleavage of the NF-κB essential modulator (NEMO), resulting in attenuation of the innate immune response to virus infection [108,109]. PDCoV nsp5 suppresses IFN expression via the cleavage of NEMO [110]. Further investigation showed that nsp5 can also cleave STAT2 but not JAK1, tyrosine kinase 2 (TYK2), STAT1, or IRF9 [111]. It is thought that NEMO probably represents a key target for 3CLpro from different coronaviruses. A structural comparison revealed that (R)-16, an inhibitor of SARS 3CLpro, also has an inhibitory effect against PEDV 3CLpro, despite PEDV 3CLpro and SARS 3CLpro sharing a sequence homology of only 45.4% [112]. Analysis of the crystal structure revealed that 3CLpro has a striking degree of conservation of the substrate-binding sites among coronaviruses, and thus, this proteinase is an ideal drug target for the treatment of diseases caused by coronavirus infection [113,114].

#### 4.3.4. Modification of the 5′-Cap Structure to Circumvent Innate Immune Recognition

Eukaryote cells assemble their mRNAs through a translation apparatus, and the 5′terminus structures of host RNA are normally modified with N7-methylation and 2′-O-methylation using enzymes during the transcription process. Viral RNAs without a 5′cap structure or methylation should be recognized by innate immune sensors [115]. RNA viruses have evolved with quite diverse mechanisms to achieve protection from the immune recognition system. Coronaviruses can produce mRNA with cap-structures themselves by enzymatic functions in their polymerase complexes [116]. It has been demonstrated that coronaviruses contain at least three capping enzymes in their polymerase complexes, including RNA triphosphatase encoded in nsp13 (NTPase helicase), N7 methyltransferase (N7-MTase) encoded in nsp14, and 2′-O-methyltransferase (2′-O-MTase) encoded in nsp16 [117]. These enzymes provide viral RNA equipped with 2′-O-methylation or N7-methylation, which gives them similar cap-structures to the host cell mRNA and subsequently suppresses the activation of TLR3/TRIF and RIG/MDA5/IPS-1 signal pathways [118]. The 2′-O-methylation of 5′cap modification can circumvent antiviral innate responses via evasion of IFIT-mediated suppression [119]. Consistently, PEDV nsp16 inhibits the expression of the *IFIT1*, *IFIT2*, and *IFIT3* genes [120], which might be associated with the 2′-O-methylation modification of nsp16.

Moreover, coronavirus proteins, such as S, nsp1, 3, 5, and 16 can suppress IFN expression, as mentioned above, and other nonstructural proteins also have multiple enzymatic functions that might impede IFN signaling, including superfamily-1 helicase activity (HEL1, nsp13), exoribonuclease activity (ExoN homolog, nsp14), and endoribonuclease activity (XendoU homolog, nsp15) [117]. In addition, the structural proteins of coronaviruses, such as M and N, exert effects on multiple levels that suppress IFN signal cascades and IFN production [121].

## 5. Therapeutic Approaches for Treatment of COVID-19

Viral replication may be particularly active during the early phrase of SARS-CoV-2 infection; thus, antiviral therapy could exerts its greatest effects before the illness progresses into the systemic hyperinflammatory stage [122]. Several antiviral drugs (remdesivir, chloroquine, and hydroxychloroquine) have been shown to have an inhibitory effect on controlling viral infection and replication in patients with COVID-19 [123]. Potential drugs may inhibit viral entry and replication via targeting the ACE2 receptor and TMPRSS2, viral membrane fusion and endocytosis, or the 3CLpro activity of SARS-CoV-2 and the RNA-dependent RNA polymerase [124].

Given the host inflammatory response to SARS-CoV-2, immune-based therapy is recommended for the treatment of moderate to severe cases of COVID-19 [123]. Immunomodulatory agents are used as therapy for dysregulated innate immune responses to SARS-CoV-2 infection. Currently recommended agents include corticosteroids, interleukin inhibitors, IFNs, and tyrosine kinase inhibitors [123,125,126]. In clinical trials, combination therapies are used for the treatment of COVID-19. For instance, patients treated with a triple combination of IFNβ-1b, lopinavir-ritonavir, and ribavirin showed faster viral clearance and more rapid clinical improvement compared with the control group [127]. A summary of the action of these agents against SARS-CoV-2 infection is shown in Table 1.

Besides the pharmacologic interventions, vaccines are an effective approach for the prevention and prophylaxis of SARS-CoV-2 [128]. The main strategies used in the development of a coronavirus vaccine involve the spike protein. Currently, there are thirty-one candidate vaccines under clinical evaluation, and six candidate vaccines have been pursued in Phase 3 clinical trials. These candidate vaccines include several standard platforms of inactivated vaccines, live-attenuated vaccines, and protein subunit vaccines, as well as some novel approaches, such as DNA-and RNA-based platforms, and replicating and nonreplicating vector platforms [129].

## 6. Conclusions and Further Pespective

In summary, SARS-CoV, MERS-CoV, SARS-CoV-2, PEDV, and PDCoV are coronaviruses that have caused pandemic diseases in humans and animals, resulting in severe public health concerns and large economic losses. These coronaviruses are typical strains that can infect mammals and cause illnesses associated with their vial tropism in the respiratory tract and/or intestinal tract. A myriad of epithelial cells and innate immune cells exert a set of robust antiviral defenses as part of the sophisticated mucosal innate immunity through cellular responses, of which IFNs and ISGs are the most fundamental molecules involved in innate immunity against coronavirus infection. However, coronaviruses are the largest types of RNA virus and have evolved with tricky strategies to evade innate immune surveillance, such as hijacking the antigen-presenting cells, shielding RNA intermediates in the replication organelle, and inhibiting the IFN signal pathway. The evasion strategies on the spike glycoprotein, nsp3 protease of PLpro, and nsp5 protease of 3CLpro of the novel SARS-COV-2 might be appealing targets for the development of antiviral agents and vaccines.

However, the biology of SARS-CoV-2 and its interactions with its host are poorly understood. Currently, no vaccines or specific drugs have been approved to prevent or treat COVID-19. It is well-known that the highly pathogenic coronaviruses can delay IFN production during the early phase of viral infection and induce hyperactive inflammatory responses in the advanced stages, which leads to the pathogenesis of these viruses. Therefore, it is very important to investigate the immunopathogenesis of SARS-CoV-2, particularly the coronavirus−host interaction and its pathogenesis. Novel three-dimensional models of tissue organoids are close to the real scenario in the respiratory and intestinal tracts and can be applied in vitro instead of as a single-cell culture model. Moreover, integrated multiomics analysis, including single-cell RNA sequencing, metagenomics, metatranscriptomics, metaproteomics, metabolomics, and other new techniques, may provide new methods that give a comprehensive understanding of the interaction of pathogens with their hosts and the microbiota.

## Figures and Tables

**Table 1:** Potential antiviral and anti-inflammatory agents under evaluation for treatment of COVID-19.

Classification	Drug Name	Mechanism of Action	Ref.
Antiviral agents	Arbidol	Binds Spike protein/ACE2 Inhibits membrane fusion of the viral envelope	[130]
	Nafacamostat mesylate	Inhibits TMPRSS2 Blocks the spread and pathogenesis of SARS-CoV	[131]
	Chloroquine Hydroxychloroquine	Inhibit viral entry and endocytosis Immunomodulatory effects	[132,133]
	Lopinavir/Ritonavir	Inhibits coronavirus 3CLpro activity	[134]
	Remdesivir Ribavirin Favipiravir	Binds viral RdRp, which inhibits viral replication via premature termination of RNA transcription	[124,132]
Corticosteroids	Dexamethasone Methylprednisolone	Potential prevention or mitigation of the systemic inflammatory responses in severe cases of COVID-19	[135]
IFNs	IFN-α/β	Binds to IFNAR1/IFNAR2 which is expressed on numerous cell types Induces transcription of ISGs	[71,72]
	IFN-λ	Binds to IFNALR1/IL10R2 in epithelial cells and some immune cells Induces transcription of ISGs	[70]
IL-1 inhibitors	Anakinra	Anti-IL-1 receptor antagonist	[125]
IL-6 inhibitors	Sarilumab	Human recombination monoclonal antibody IL-6 receptor antagonist	[136]
	Tocilizumab	Recombinant humanized monoclonal antibody IL-6 receptor antagonist	[137]
JAK inhibitors	Baricitinib	JAK inhibitor selective for JAK1, JAK2, and TYK2 Theoretical antiviral properties via inhibition of AKK1 that may prevent viral entry and infection Inhibition of IL-6 induced STAT3 phosphorylation	[138]
	Ruxolitinib	JAK inhibitor selective for JAK1and JAK2 Theoretical antiviral activities via inhibition of AKK1 that may prevent viral entry and infection Inhibition of IL-6 through JAK1/JAK2 pathway	[139]

## Abbreviations

Ref.	references
IFN	interferons
IL-1	interleukin-1
IL-6	interleukin-6
JAK	Janus kinase
ACE2	angiotensin-converting enzyme 2
TMPRSS2	transmembrane serine protease 2
3CLpro	3-chymotrypsin-like protease
RdRp	RNA-dependent RNA polymerase
STAT	signal transducer and activator transcription
TYK2	tyrosine kinase 2
AKK1	adaptor-associated kinase-1
